# Detecting racial and ethnic disparities in study exclusion: screening outcomes from a RCT for pregnant women with insomnia

**DOI:** 10.1186/s13063-026-09422-y

**Published:** 2026-01-13

**Authors:** Carolyn Ponting, Candance Sorensen, Bernadette McClelland, Richelle Mah, John Neuhaus, Rachel Manber, Andrew D. Krystal, Patricia Moran, Jennifer N. Felder

**Affiliations:** 1 Department of Psychiatry and Behavioral Sciences, University of California, San Francisco, San Francisco, USA; 2 Osher Center for Integrative Health, University of California, San Francisco, San Francisco, USA; 3 Department of Counseling Psychology and Human Development, University of Oregon, Eugene, USA; 4 Department of Epidemiology and Biostatistics, University of California, San Francisco, San Francisco, USA; 5 Department of Psychiatry and Behavioral Sciences, Stanford University, Palo Alto, USA

**Keywords:** Recruitment, Race, Ethnicity, Clinical trial, Eligibility criteria, Exclusion

## Abstract

**Background:**

Eligibility criteria are a critical component of a well-designed clinical trial, enhancing trial safety and internal validity. Yet, data suggest that exclusion rates based on these criteria often vary by participant race/ethnicity.

**Method:**

This study compared the proportion of participants (*n* = 4235) from seven racial/ethnic groups, who were included versus excluded from participation in a randomized controlled trial (RCT) testing two digital sleep interventions for the prevention of perinatal depression. Eight 2 × 7 chi-squared tests were conducted to compare the proportion of each racial/ethnic group excluded due to each eligibility criterion. Logistic regressions were fitted to estimate the magnitude of the relationship between racial/ethnic group and exclusion based on each eligibility criterion.

**Results:**

The proportion of excluded participants differed by race/ethnicity across all eight eligibility criteria. For example, Black participants were more likely to be excluded due to comorbid conditions such as sleep apnea *X*^2^ (6, *N* = 4151) = 20.94, *p* = .002, and Asian participants were more likely to be excluded for reporting subclinical insomnia symptoms *X*^2^ (6, *N* = 4151) = 85.99, *p* < .001. Logistic regressions showed that compared to White participants, Black participants had significantly higher odds (odds ratios ranging from 1.70 to 6.86) of study exclusion for three of the eight eligibility criteria.

**Conclusions:**

Eligibility criteria excluded prospective study participants at different rates dependent on their race/ethnicity. Differences in trial exclusion can contribute to the under-enrollment of minoritized pregnant people in RCTs for behavioral health. Quantifying and reporting eligibility disparities enables investigators to more precisely evaluate the trade-offs of specific inclusion criteria against the generalizability of findings to diverse populations.

## Racial and ethnic differences in study exclusion from a CBT-I trial to prevent perinatal depression

### Background

Randomized controlled trials (RCTs) are undertaken to establish a causal link between an intervention and clinical outcome (i.e., intervention efficacy). While RCTs are considered the gold standard for evaluating the effects of an intervention, they have long been criticized for failing to adequately enroll diverse samples, including racial and ethnic minorities [1–4]. These critiques are of continued relevance. A review of US-based clinical trials registered in ClinicalTrials.gov from 2000 to 2020, consisting of 20,692 studies, showed that only 43% of trials reported the racial or ethnic makeup of their participant samples [5]. Of the trials that did report race and ethnicity, 80% of participants identified as non-Latinx white [5]. In addition to intervention samples being disproportionately non-Latinx white [6–8], they are also more likely to be of middle to high socioeconomic status [9, 10], and less likely to present with medical or mental health comorbidities [11, 12]. Data from these relatively homogenous participant pools make up the majority of our intervention evidence base, but do not reflect the broader population and thus, clinical guidelines and areas of clinical innovation may lack generalizability for minoritized groups [5, 13].

There are several contributors that lead to relatively homogenous participant pools in RCTs for behavioral health interventions [2]. Notably, recruitment challenges are commonplace. Many researchers rely on convenience samples or are met with limited buy-in from clinics and community partners, who are rarely compensated for their recruitment efforts and are juggling competing demands [14, 15]. Participants from minoritized backgrounds may also feel warranted skepticism about participating in intervention research given historical abuses by researchers, or may face logistical challenges to participation due to language barriers or time constraints associated with disproportionate caregiving burdens and access to reliable transportation [16, 17]. Less studied, however, are trial design characteristics—like inclusion and exclusion criteria—that can inadvertently keep minoritized people from participating at disproportionate rates [1].

Inclusion and exclusion criteria aim to enhance trial safety and internal validity. Criteria are selected to ensure that participants receiving an intervention have the clinical presentation and severity most likely to benefit from treatment and that additional health conditions or medications do not blunt treatment effects [18]. However, disproportionate exposure to adverse social determinants of health like poverty, racism, access to health care, and safe housing means that ethnic/racial minorities and low-income groups are more likely to have health conditions and life circumstances that commonly exclude them from trial participation [19, 20].

For example, in a smoking cessation study that used purposeful sampling and stratified their randomization so that an equal number of Black, Latina, and non-Latina White participants would be assigned to each treatment arm—study exclusion criteria disproportionately impacted Black and Latina participants [21]. Webb Hooper and colleagues [21] reported that while non-Latina White participants were ineligible at a rate of 24%, Latina participants were ineligible at a rate of 37%, and Black participants at a rate of 42%—differences that were driven by higher rates of exclusionary mental health comorbidities. Aligned with these findings, other smoking-cessation and cancer trials have found that Black prospective participants were disproportionately excluded from trial participation due to residential instability (i.e., not having a home address at intake) [22], or medical comorbidities [23]. Taken together, available data suggest that exclusion criteria do not have equal impacts on prospective participants.

While eligibility criteria may be modified to enhance trial diversity, certain exclusion criteria are well justified and necessary even when they impact groups at different rates. Thus, examining whether racially or ethnically minoritized groups or low-income groups are disproportionately impacted by exclusion criteria can identify demographic groups that ought to be screened at greater numbers to enhance the likelihood of obtaining a representative sample. The present study examined whether the rates of exclusion in an RCT evaluating two digital sleep interventions for the prevention of perinatal depression were different across several distinct criteria, dependent on prospective participants’ race or ethnicity. Findings provide insight into the design characteristics that can contribute to homogenous samples in randomized trials and unintentionally perpetuate a clinical evidence base that lacks generalizability.

### Methods

#### Participants

Participants in this study were pregnant people (*n* = 4235) who took an online screening survey to determine their potential eligibility for a randomized controlled trial testing the efficacy of two sleep interventions—digital cognitive behavioral therapy for insomnia (CBT-I) and Sleep Hygiene Education (SHE)—for the prevention of perinatal depression. The clinical trial is registered at Clinicaltrial.gov (Protocol: NCT05596318, uploaded 10/24/2022). Participants were recruited nationwide through advertisements on Meta (i.e., Facebook, Instagram) and two popular pregnancy planning apps, BabyCenter and What to Expect. Over half of prospective participants (*n* = 2340; 55.3%) were excluded based on the results of this initial screener. The present study reports on differences in study exclusion among participants who took the online screening between November 2022 and August 2024.

#### Design

Prospective participants who clicked on the online study advertisements were directed to a study landing page that provided a brief summary of the study goals and procedures, and a detailed screening consent form. Prospective participants who signed the screening consent form were directed to complete the screening survey. All screening procedures were approved by the Institutional Review Board at the University of California, San Francisco, IRB # 21–35440. The first screening questions asked participants whether they were currently pregnant and, if so, asked for their gestational age and due date. Participants between 14 and 25 weeks pregnant were invited to continue with the screening survey. See Table 1 for eligibility questions, response options, and rationale for each screening criterion. In addition to responding to eligibility questions, prospective participants were also asked to provide basic demographic data. This included their age, household income, number of people supported by their household income, educational attainment, zip code of residence, and race/ethnicity. The present study used U.S. Census Bureau definitions of race and ethnicity. Race was defined as a person’s self-identification with one or more social groups based on physical characteristics, ancestry, or shared history; these categories are understood to represent social constructions as opposed to biological or genetic classifications. Ethnicity was defined as an individual’s self-identification of the origin of their nationality, culture, or language, and is distinct from race. Participants indicated their racial identity by responding to the question: “Which of the following categories best describes your race? (Select all that apply) (i.e., American Indian or Alaskan Native, Asian, White, Black or African American, Native Hawaiian or Other Pacific Islander, A race not described here).” Participants indicated their ethnicity by responding to the question: “Are you of Hispanic, Latino/a/x or Spanish origin? (i.e., yes, no).”

**Table 1:** Eligibility survey items administered to prospective participants and their rationale

Question	Response options	Eligible response	Exclusion rationale
Are you currently on bed rest?	A. Yes B. No	No	Participants placed on bedrest would not be able to engage in the time in bed restriction component of CBT-I
What is your age (in years)?	Open text	≥ 18	Adolescents have unique sleep needs
Do you have daily access to a computer, phone, or tablet that is connected to the internet?	A. Yes B. No	Yes	Interventions are delivered digitally
Are you able to read and speak English?	A. Yes B. No	Yes	The digital CBT-I app is currently available in English only
Has a healthcare provider every diagnosed you with bipolar disorder, or told you that you were manic or hypomanic?	A. Yes B. No	No	The time in bed restriction component of CBT-I could precipitate or exacerbate symptoms of mania
Have you ever experienced psychosis (hallucinations or delusions) or been diagnosed with a psychotic disorder, like schizophrenia?	A. Yes B. No	No	Symptoms of psychosis could be exacerbated by the time in bed restriction component of CBT
Has a healthcare professional ever diagnosed you with any of the following sleep disorders? Check all that apply	A. Insomnia disorder B. Sleep apnea C. Narcolepsy D. Parasomnia E. Circadian rhythm disorder F. Restless leg syndrome G. None of the above	Insomnia disorder OR None of the above	Exclusionary sleep disorders are unlikely to benefit from digital CBT-I
Do you work the night shift?	A. Yes B. No	No	CBT-I would likely be ineffective or the effect would be difficult to interpret
Are you currently taking an antidepressant? (e.g., SSRI, SNRI, TCA, MAOI)	A. Yes B. No C. Not sure	No OR Not sure	Antidepressant medication use would make the effect of CBT-I on preventing depression difficult to interpret
Are you planning to begin taking an antidepressant before you are 12 months postpartum?	A. Yes B. No C. Not sure	No OR Not sure	Antidepressant medication use would make the effect of CBT-I on preventing depression difficult to interpret

#### Data analysis

The objective of the statistical analysis was to compare the proportion of each racial/ethnic group excluded due to each eligibility criterion. For the purposes of these analyses, seven ethnic and racial categories were compared: (a) Black, (b) American Indian/Alaska Native/Native Hawaiian or Other Pacific Islander (AI/AN/NHPI), (c) Asian, (d) White, (e) Multiracial (i.e., individuals who selected more than one race), (f) Latina (i.e., inclusive of individuals who indicated their race was White or “other” as well as Latino/a/x ethnicity), and (g) Latina Black (i.e., individuals who reported Black race and Latino/a/x ethnicity). A 2 × 7 chi-squared test was conducted to compare the proportion of each racial/ethnic group excluded due to each of the eight eligibility criteria. All eight exclusion criteria were coded dichotomously (i.e., yes, no); the criteria assessed were:

- Being on bedrest,
- A self-reported history of mania or bipolar disorder,
- A self-reported history of sleep apnea,
- A self-reported history of restless leg syndrome,
- Current night shift work,
- Current antidepressant medication use,
- Planned antidepressant use prior to the first year postpartum, and
- Subclinical insomnia severity scores (i.e., score < 11 on the Insomnia Severity Index [24]).

While each exclusion criterion was examined independently, participants excluded at the screening stage may have been disqualified from participating for multiple reasons. An additional 2 × 7 chi-squared test was conducted to assess whether there were differences in the proportion of each racial or ethnic group who were excluded for multiple reasons compared to those who were not.

To assess the magnitude of the independent associations between each racial/ethnic group and the likelihood of being excluded by each eligibility criterion, we fitted eight binary logistic regression models. The same racial/ethnic groups tested in the chi-squared analyses were dummy coded and entered as independent variables, with White participants serving as the reference group. The dependent variable was study exclusion, where study inclusion served as the reference group. About 2% of participants (*n* = 84) did not report race or ethnicity data and was not included in chi-squared or logistic regression analyses, leaving a sample of 4151.

We did not adjust models for income or education because the goal of the study was to document and quantify the extent to which trial eligibility criteria differentially impact racial/ethnic groups. Socioeconomic variables are themselves shaped by structural racism and systemic inequities; therefore, accounting for them in regression models has the potential to underestimate the effect of racial/ethnic structural disadvantage [25]. Moreover, from a practical standpoint, unadjusted models may offer greater utility for clinical trialists who are unlikely to have access to recruitment pools in which socioeconomic status is evenly distributed across racial/ethnic groups. Systemic inequities in the USA have shaped the economic and educational attainment of racially/ethnically minoritized populations, contributing to persistent disparities across these domains [26, 27]. Data were analyzed using IBM SPSS (version 29) software.

## Results

**Fig. 1:**
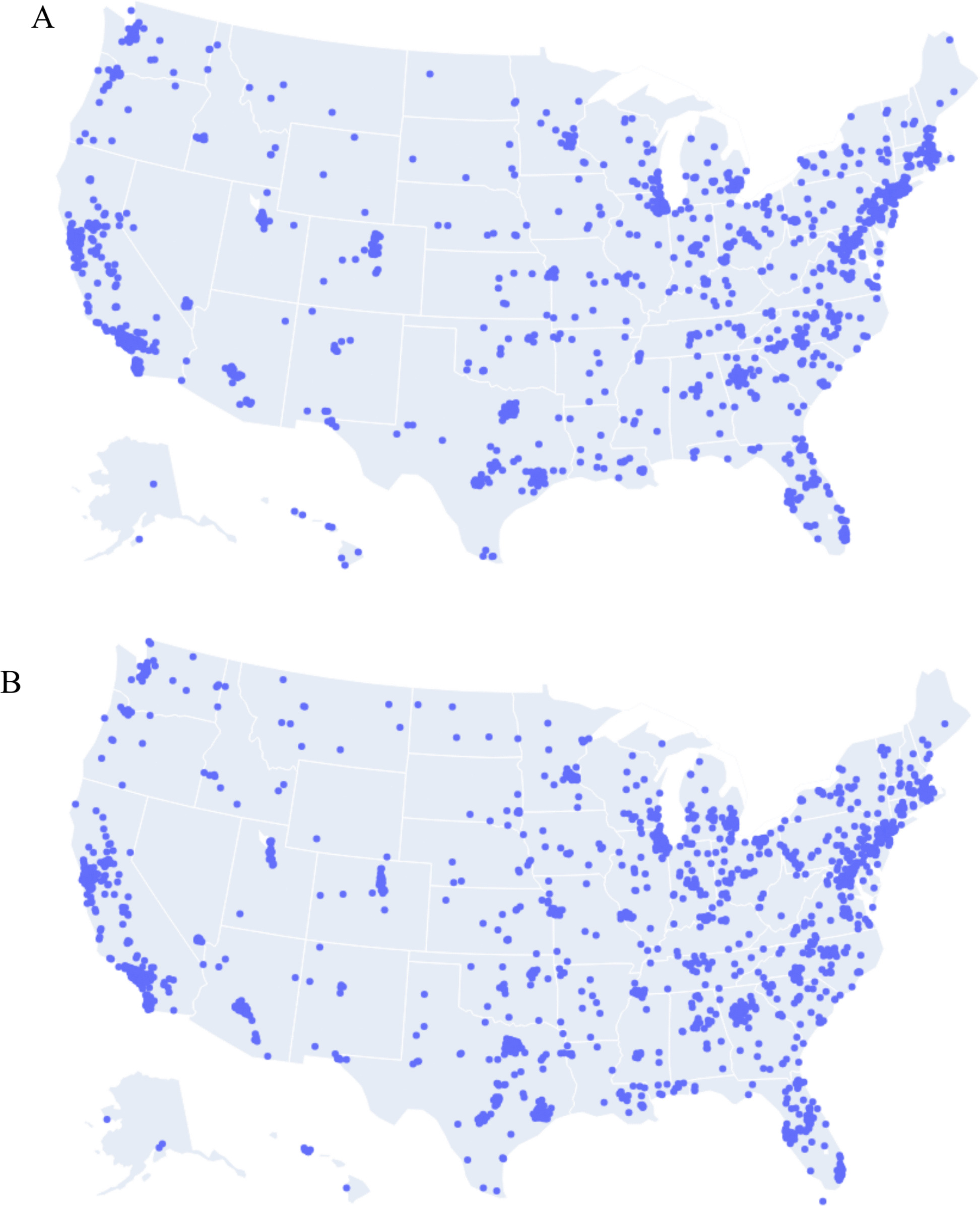
Location of included (top; panel **A**) and excluded (bottom; panel **B**) participants plotted by zip code

For detailed demographic characteristics of included and excluded participants, see Table 2 and Fig. 1. Overall, the proportion of participants excluded from the study at screening differed depending on race and ethnicity *X*^2^ (df = 6, *N* = 4151) = 17.23, *p* = 0.008. Latina participants were least likely to be excluded (48.0%), while Multiracial participants were most likely to be excluded (60.4%). The proportion of participants who were excluded due to multiple reasons differed by racial or ethnic group *X*^2^ (6, *N* = 4151) = 28.146, *p* < 0.001. Multiracial (21.7%) and Black participants (21.4%) were most likely to be excluded for multiple reasons, while American Indian/Alaska Native/Native Hawaiian and Pacific Islander (11.5%) and Asian participants (12.1%) were least likely to be excluded for multiple reasons. Table 3 compares the proportion of participants from each racial/ethnic group who were eligible or ineligible across each criterion.

**Table 2:** Sample Characteristics of prospective participants who were included versus excluded following initial screening survey

	Included (*n*, %)	Excluded (*n*, %)	Chi-squared
Race/ethnicity			*X*^2=^17.23, *p* =.008
Asian (*n* = 338)	153 (45.3%)	185 (54.7%)	
American Indian, Alaska Native, Native Hawaiian or Other Pacific Islander (*n* = 78)	38 (48.7%)	40 (51.3%)	
Black (*n* = 739)	327 (44.2%)	412 (55.8%)	
Latina Black (*n* = 71)	36 (50.7%)	35 (49.3%)	
Latina (*n* = 667)	347 (52.0%)	320 (48.0%)	
Multiracial* (*n* = 217)	86 (39.6%)	131(60.4%)	
White (*n* = 2041)	904 (44.3%)	1,137 (55.7%)	
Total family income before taxes			*X*^2=^22.16, *p* <.001
Less than $25,000 (*n* = 461)	169 (36.7%)	292 (63.3%)	
$25,000–$49,999 (*n* = 650)	285 (43.8%)	365 (56.2%)	
$50,000–$99,999 (*n* = 1028)	508 (49.4%)	520 (50.6%)	
$100,000–$199,999 (*n* = 1161)	535 (46.1%)	626 (53.3%)	
$200,000 or more (*n* = 674)	315 (46.7%)	359 (53.3%)	
Don’t know/prefer not to say (*n* = 266)	86 (32.3%)	180 (67.6%)	
Education			*X*^2=^32.10, *p* <.001
Less than a high school degree (*n* = 81)	20 (24.7%)	61 (75.3%)	
High school graduate/GED (*n* = 451)	451 (39.5%)	273 (60.5%)	
Some college (*n* = 904)	423 (46.8%)	481 (53.2%)	
Bachelor’s degree (*n* = 1,316)	665 (49.8%)	661 (50.2%)	
Professional/graduate degree (*n* = 1403)	619 (44.1%)	784 (55.9%)	
Relationship status			*X*^2^ = 3.18, *p* =.20
Married/living with partner (*n* = 3669)	1649 (44.9%)	2020 (55.1%)	
Partnered, living separately (*n* = 295)	138 (46.8%)	157 (53.2%)	
Single, not involved with partner (*n *= 271)	108 (39.9%)	163 (60.1%)	

*Multiracial participants included 59.4% of participants who identified as Black and another race and 37.8% that identified as American Indian/Alaska Native and another race

**Table 3:** Frequencies and percentages of participants (*n* = 4145) from each racial/ethnic group excluded from study participation based on each eligibility criterion

	Black	AI/AN/NHPI	Asian	White	Multi-racial	Latina	Latina Black
Bed rest							
Yes	82 (11.1%)	1 (1.3%)	16 (4.7%)	28 (1.4%)	9 (4.1%)	27 (4.0%)	3 (4.2%)
Lifetime history of bipolar disorder or mania							
Yes	53 (7.2%)	7 (9.0%)	4 (1.2%)	113 (5.5%)	21 (9.7%)	28 (4.2%)	9 (12.7%)
Lifetime history of sleep apnea							
Yes	45 (6.1%)	1 (1.3%)	10 (3.0%)	70 (3.4%)	14 (6.5%)	18 (2.7%)	5 (7.0%)
Lifetime history of restless leg syndrome							
Yes	18 (2.4%)	2 (2.6%)	2 (0.6%)	98 (4.8%)	7 (3.2%)	15 (2.2%)	1 (1.4%)
Current night shift work							
Yes	58 (7.8%)	3 (3.8%)	10 (3.0%)	58 (2.8%)	15 (6.9%)	29 (4.3%)	4 (5.6%)
Current antidepressant medication use							
Yes	55 (7.4%)	8 (10.3%)	27 (8.0%)	390 (19.1%)	24 (11.1%)	65 (9.7%)	3 (4.2%)
Planned antidepressant medication use							
Yes	66 (8.9%)	7 (9.0%)	18 (5.3%)	265 (13.0%)	21 (9.7%)	47 (7.0%)	4 (5.6%)
Subclinical insomnia severity score							
Yes	95 (12.9%)	8 (10.3%)	117 (34.6%)	437 (21.4%)	46 (21.1%)	124 (18.6%)	4 (5.6%)
Total	739 (100%)	78 (100%)	338 (100%)	2041 (100%)	217 (100%)	667 (100%)	71 (100%)

*AI/AN/NHPI* American Indian, Alaska Native, Native Hawaiian or Other Pacific Islander

Table 4 presents results from eight logistic regressions, showing odds ratios, and 95% confidence intervals, for exclusion likelihood by eligibility criterion across racial and ethnic groups. Regarding eligibility criteria that assessed for health concerns and comorbidities that may have impacted the cost-benefit analysis of the sleep programs, exclusion differed by race and ethnicity. For example, the proportion of participants excluded due to bedrest differed by race or ethnicity *X*^2^ (6, *N* = 4,151) = 135.65, *p* < 0.001. Logistic regressions demonstrated that compared to their White counterparts**,** Black participants had over six times the odds of being excluded due to current bedrest (OR = 6.86), while Asian (OR = 2.73), Multiracial (OR = 2.37), and Latina (OR = 2.32) women had more than twice the odds of exclusion for the same reason. The proportion of participants excluded due to self-reported history of mania or bipolar disorder *X*^2^ (6, *N* = 4151) = 33.28, *p* < 0.001, and self-reported history of sleep apnea* X*^2^ (6, *N* = 4151) = 20.94, *p* = 0.002 also differed by participant race or ethnicity. Logistic regressions demonstrated that compared to their White counterparts, Latina Black participants had 2.42 times the odds and Multiracial participants had 1.74 times the odds of being excluded due to self-reporting a history of mania or bipolar disorder, while Asian participants had one fifth the odds (OR = 0.20) of being excluded for the same reason. Multiracial participants had 1.81 times the odds and Black participants 1.70 times the odds of being excluded due to self-reporting a history of sleep apnea compared to their White counterparts.

**Table 4:** Odds ratios and 95% confidence intervals assessing the magnitudes of association of participant race/ethnicity with study exclusion for each eligibility criterion

	Odds Ratio	95% CI		
		LL		UL
**Bed rest**				
Omnibus test	$\chi^2$[6,4235] = 102.55, *p* <.001			
White	1.00			
AI/AN/NHPI	.71	.10		5.26
**Asian**	**2.73**	**1.50**		**4.95**
**Black**	**6.86**	**4.62**		**10.17**
**Multiracial**	**2.37**	**1.13**		**4.98**
**Latina**	**2.32**	**1.40**		**3.83**
Latina Black	2.42	.73		8.04
**Lifetime history of bipolar disorder or mania**				
Omnibus test	$\chi$^2^(6, 4235) = 36.01, *p* <.001			
White	1.00			
AI/AN/NHPI	1.61	.72	3.56	
**Asian**	**.20**	**.07**	**.53**	
Black	1.26	.90	1.76	
**Multiracial**	**1.74**	**1.07**	**2.83**	
Latina	.71	.47	1.09	
**Latina Black**	**2.36**	**1.15**	**4.87**	
**Lifetime history of sleep apnea**				
Omnibus test	$\chi$^2^(6, 4235) = 18.55, *p* <.005			
White	1.00			
AI/AN/NHPI	.34	.05	2.48	
Asian	.80	.41	1.56	
**Black**	**1.70**	**1.17**	**2.48**	
**Multiracial**	**1.81**	**1.01**	**3.26**	
Latina	.73	.43	1.22	
Latina Black	1.99	.78	5.07	
**Lifetime history of restless leg syndrome**				
Omnibus test	$\chi$^2^(6, 4235) = 34.31, *p* <.001			
White	1.00			
AI/AN/NHPI	.49	.12	2.03	
**Asian**	**.11**	**.03**	**.45**	
**Black**	**.47**	**.28**	**.77**	
Multiracial	.62	.29	1.36	
**Latina**	**.43**	**.25**	**.74**	
Latina Black	.27	.04	1.94	
**Current night shift work**				
Omnibus test	$\chi$^2^(6, 4235) = 27.54, *p* <.001			
White	1.00			
AI/AN/NHPI	1.14	.35	3.70	
Asian	.87	.44	1.70	
**Black**	**2.43**	**1.70**	**3.47**	
**Multiracial**	**2.12**	**1.19**	**3.76**	
Latina	1.30	.84	2.01	
Latina Black	1.70	.60	4.80	
**Current antidepressant medication use**				
Omnibus test	$\chi$^2^(6, 4235) = 105.90, *p* <.001			
White	1.00			
AI/AN/NHPI	.48	.23	1.01	
**Asian**	**.37**	**.24**	**.55**	
**Black**	**.34**	**.25**	**.46**	
**Multiracial**	**.53**	**.34**	**.82**	
**Latina**	**.46**	**.35**	**.60**	
**Latina Black**	**.19**	**.06**	**.60**	
**Planned antidepressant medication use**				
Omnibus test	$\chi$^2^(6, 4235) = 39.05, *p* <.001			
White	1.00			
AI/AN/NHPI	.66	.30	1.44	
**Asian**	**.38**	**.23**	**.61**	
**Black**	**.65**	**. 49**	**.87**	
Multiracial	.72	.45	1.14	
**Latina**	**.51**	**.37**	**.70**	
Latina Black	.40	.14	1.10	
**Subclinical Insomnia Severity Score**				
Omnibus test	$\chi$^2^(6, 4235) = 84.90, *p* <.001			
White	1.00			
**AI/AN/NHPI**	**.43**	**.21**	**.90**	
**Asian**	**2.00**	**1.56**	**2.56**	
**Black**	**.56**	**.44**	**.71**	
Multiracial	1.02	.72	1.43	
Latina	.86	.69	1.08	
**Latina Black**	**.23**	**.08**	**.62**	

*AI/AN/NHPI* American Indian, Alaska Native, Native Hawaiian or Other Pacific Islander

Bolded effects are significant at a threshold of *p* <.05. The reference group is White

Some participants were excluded due to working conditions that would have required specific adaptations to the sleep intervention. The proportion of participants excluded due to current night shift work *X*^2^ (6, *N* = 4151) = 38.88, *p* < 0.001 differed among racial or ethnic groups. A logistic regression demonstrated that compared to White participants, Black participants had 2.43 times the odds of exclusion due to night shift work, while Multiracial participants had 2.12 times the odds of being excluded for the same reason.

Participants were also excluded for antidepressant medication use, which would have obscured preventive effects of the interventions on depression. The proportion of participants excluded due to current antidepressant medication use *X*^2^ (6, *N* = 4151) = 100.06, *p* < 0.001, and planned antidepressant use prior to the first year postpartum* X*^2^ (6, *N* = 4151) = 35.99, *p* < 0.001, differed based on participant race or ethnicity. Logistic regressions demonstrated that Latina Black (OR = 0.19), Black (OR = 0.34), Asian (OR = 0.37), Latina (OR = 0.46), and Multiracial (OR = 0.53) participants all had reduced odds of exclusion due to current antidepressant medication use compared to their White counterparts. Asian (OR = 0.38), Latina (OR = 0.51), and Black (OR = 0.65) participants also had reduced odds of exclusion due to reported plans to begin antidepressant medication prior to the first year postpartum compared to their White counterparts.

Finally, many participants were excluded because their insomnia symptoms were subclinical, which would have limited the detection of symptom improvement. The proportion of participants excluded due to subclinical severity scores differed by race and ethnicity *X*^2^ (6, *N* = 4151) = 85.99, *p* < 0.001. A logistic regression demonstrated that compared to their White counterparts, Asian women had 2.00 times the odds of exclusion due to subclinical severity scores on a validated insomnia screening measure, while Black (OR = 0.56), Latina Black (OR = 0.23), and AI/AN/NHPI (OR = 0.43) participants had reduced odds of exclusion for the same reason.

## Discussion

The present study examined racial/ethnic differences in eligibility for a randomized trial testing two digital sleep interventions for the prevention of perinatal depression. Overall, Multiracial participants (a majority of whom identified as Black and another race) were excluded at the highest rates following initial screening, while Latina participants were excluded at the lowest rates. Further, across all eight eligibility criteria assessed, participants were excluded at different rates based on their race or ethnicity, and Multiracial and Black participants were most likely to be excluded for multiple reasons. Multiracial and Black participants had significantly higher odds (odds ratios ranging from 1.70 to 6.86) of study exclusion across three of the eight eligibility criteria (i.e., bed rest, night-shift work, and lifetime sleep apnea). These data suggest that even among individuals demonstrating interest in participating in a clinical trial for prenatal insomnia, eligibility criteria can exclude participants of particular racial/ethnic identities at different rates.

Compared to other racial and ethnic groups, Black and Multiracial participants—most of whom identified as Black—had increased odds of being excluded due to comorbid conditions such as sleep apnea or prescribed bed rest. A wide range of clinical trials, including those that test non-surgical treatments for cancer [28], rheumatoid arthritis [29], and cardiovascular disease [30], also report high rates of exclusion of Black participants due in part to comorbid health conditions. Inequities, including systemic racism, socioeconomic disparities, and poorer quality health care [31, 32], contribute to the high rates of mental and physical comorbidities experienced by Black communities in the USA [31, 33]. These findings highlight trial design features that may unintentionally exclude Black participants. Specifically, the greater the number of exclusionary criteria based on co-morbid mental and physical health conditions, the higher the likelihood that Black participants will be excluded.

This study showed that a larger proportion of Latina Black participants reported a history of bipolar disorder or sleep apnea than any other racial or ethnic group. Further, logistic regressions revealed that Latina Black participants had significantly greater odds of exclusion due to a history of bipolar disorder compared to White participants. While Latina ethnicity is often examined without attention to race, preliminary evidence suggests that Latina Black (also referred to as Afro-Latina) women have worse maternal health and birth outcomes than their White or Latina counterparts [34]. Disparities in health outcomes are partly driven by the compounded social stressors such as discrimination associated with holding multiple minority identities [35, 36] and less access to healthcare. Increasingly, scholars in maternal mental health equity have encouraged race data be disaggregated from Latina ethnicity [37] to more accurately characterize the heterogeneity in Latinx communities and better understand the health needs of each subpopulation. Present findings showed differential exclusion rates between Latina and Latina-Black participants dependent on eligibility criteria. Criteria that disproportionately exclude Black women, including those of Latinx ethnicity, from perinatal intervention research warrant particular scrutiny, given that these groups face significantly higher risks of adverse maternal and infant health outcomes [38, 39] that could be mitigated by psychological intervention [40].

Black participants, and Multiracial participants were also most likely to be excluded from participation due to night shift work. While both interventions tested in the present RCT were self-guided and app-based, allowing for intervention content to be delivered at participants’ convenience, the app did not have a tailored version of insomnia intervention techniques (e.g., sleep restriction) required for shift workers. Occupational demands, particularly shift work and job-related inflexibility, are social determinants of health that disproportionately impact Black individuals and are likely to limit their ability to meet scheduling requirements for many clinical trials [41].

White participants were the racial group most likely to be excluded due to current or planned use of antidepressant medication during the study. Further, Asian, Black, and Latina participants had smaller odds of exclusion due to current or planned antidepressant medication use compared to their White counterparts. This was unsurprising; in the USA, White individuals [42], including those who are pregnant [43], use antidepressants at higher rates than non-white racial groups or those of Latinx ethnicity. Several studies have documented that ethnic and racial minorities find psychiatric medications undesirable due to stigma and concerns about drug dependency, preferring psychotherapy instead [44–47]. While the relatively high exclusion rate of White participants at screening (55.7%) was primarily due to current or planned antidepressant use, White participants were screened at significantly higher rates than any other racial or ethnic group. Thus, White participants were still the largest group represented among eligible screeners. Given the much higher rate of screening response from White women across clinical trials, it is unlikely that eligibility criteria that exclude White women at higher rates will lead to the under-enrollment of this racial group [5].

Finally, just over half of Asian participants were excluded (54.7%) at screening, most often because their insomnia symptoms were not severe enough. Despite responding to an advertisement specifically recruiting pregnant people who were struggling to sleep, compared to their White counterparts, Asian participants had 2 times the odds of exclusion due to insomnia symptoms that did not reach clinical significance. Extant research suggests that underreporting of health issues—including sleep disorders—is prevalent among Asian Americans, a pattern often attributed to cultural stigma and norms [48, 49]. Many Asian cultures emphasize stoicism and resilience, leading individuals to minimize personal struggles or avoid acknowledging them altogether [50]. Further, the “model minority” stereotype perpetuates the expectation that Asian Americans are less likely to experience health challenges, adding pressure to conform to societal ideals of success and well-being [51] and may result in under-reporting of insomnia symptoms. Additionally, there may be a need for existing insomnia screening tools to be evaluated for cultural invariance to ensure their validity across diverse groups, or to develop more sensitive screening tools. While confirmatory factor analyses of the ISI have demonstrated the screener performed equally well across White and Black participants [52], similar analyses have not been conducted with Asian participants or other racial groups.

The analysis of screening data has inherent limitations. For example, all psychiatric disorders and medical data (e.g., gestational age) were measured via self-report. At later, more intensive screening steps prior to randomization, participants provide pregnancy documentation (e.g., an ultrasound) and participate in a diagnostic clinical interview to ensure these eligibility criteria are corroborated. However, at this initial screening step, there are necessarily fewer ethically allowable ways of confirming self-reported data. The use of targeted recruitment efforts to identify pregnant women, rates of exclusion similar to those of other prenatal insomnia trials [53], and recent validation studies demonstrating that the generally high accuracy of self-reported psychiatric history [54] temper these concerns. Additionally, two of the racial/ethnic groups in our sample—Latina Black (*n* = 71) and American Indian/Alaska Native/Native Hawaiian and Pacific Islander (*n* = 72)—had substantially smaller sample sizes compared to other groups. While representation of these groups—each 1.7% of the sample—reflect national demographics, the smaller sample sizes resulted in wider confidence intervals for estimates of trial eligibility. Trials with higher screening rates of Latina Black and American Indian or Indigenous participants may be needed to achieve sufficient power to detect statistically significant differences in odds of study exclusion.

## Conclusion

While the disparities in eligibility identified in the present study can be interpreted within the context of prenatal intervention trials, the process of evaluating racial/ethnic differences in eligibility criteria has much broader implications. Differences in trial exclusion can contribute to the under-enrollment of minoritized pregnant people in RCTs for behavioral health. Examining the impact of eligibility criteria on sample diversity can help investigators weigh decisions about the rigor of their criteria against sample representativeness. When disparities in exclusion are found, trialists can assess whether the eligibility criteria are justified or if specific criteria are too strict, unintentionally leading to homogeneous samples. An additional possibility is to alter recruitment plans to better reach specific ethnic or racial groups found to be excluded at high rates [55] with the goal of enhancing screening to make up for lower conversion (i.e., eligibility) rates. Our findings and those of others [21–23] identify that Black individuals—including those who are Multiracial or Latina—are more likely to be excluded from clinical trials despite demonstrating interest. Given the persistent under-enrollment of non-White individuals [5], examining racial and ethnic differences in eligibility criteria is a data-driven strategy that can be leveraged to improve participant diversity in RCTs and promote more equitable clinical practice.

## Data Availability

The datasets during and/or analyzed during the current study are available from the corresponding author on reasonable request.

## References

[1] Hussain-Gambles M, Atkin K, Leese B. Why ethnic minority groups are under-represented in clinical trials: a review of the literature. Health Soc Care Community. 2004;12(5):382–8. 15373816 10.1111/j.1365-2524.2004.00507.x

[2] Kennedy-Martin T, Curtis S, Faries D, Robinson S, Johnston J. A literature review on the representativeness of randomized controlled trial samples and implications for the external validity of trial results. Trials. 2015;3(16):495. 10.1186/s13063-015-1023-4 PMC4632358 26530985

[3] Mulder R, Singh AB, Hamilton A, Das P, Outhred T, Morris G, et al. The limitations of using randomised controlled trials as a basis for developing treatment guidelines. Evid Based Ment Health. 2018;21(1):4–6. 28710065 10.1136/eb-2017-102701 PMC10270454

[4] Owusu-Addo E, Bennor DM, Orkin AM, Chan AW, Welch VA, Treweek S, et al. Recruitment, retention and reporting of variables related to ethnic diversity in randomised controlled trials: an umbrella review. BMJ Open. 2024;14(8):e084889. 39122387 10.1136/bmjopen-2024-084889 PMC11340254

[5] Turner BE, Steinberg JR, Weeks BT, Rodriguez F, Cullen MR. Race/ethnicity reporting and representation in US clinical trials: a cohort study. Lancet Reg Health - Am. 2022;11:100252. 10.1016/j.lana.2022.100252 PMC9302767 35875251

[6] Jesús-Romero RD, Holder-Dixon AR, Buss JF, Lorenzo-Luaces L. Race, ethnicity, and other cultural background factors in trials of internet-based cognitive behavioral therapy for depression: systematic review. J Med Internet Res. 2024;26(1):e50780. 38300699 10.2196/50780 PMC10870215

[7] Riccioni A, Radua J, Ashaye FO, Solmi M, Cortese S. Systematic review and meta-analysis: reporting and representation of race/ethnicity in 310 randomized controlled trials of attention-deficit/hyperactivity disorder medications. J Am Acad Child Adolesc Psychiatry. 2024;63(7):698–707. 37890665 10.1016/j.jaac.2023.09.544

[8] Steinbrenner JR, McIntyre N, Rentschler LF, Pearson JN, Luelmo P, Jaramillo ME, et al. Patterns in reporting and participant inclusion related to race and ethnicity in autism intervention literature: data from a large-scale systematic review of evidence-based practices. Autism. 2022;26(8):2026–40. 35068190 10.1177/13623613211072593 PMC9596958

[9] Peter SC, Pfund RA, Ginley MK. Increased demographic representation in randomized control trials for gambling disorder in the United States is needed: a systematic review. J Gambl Stud. 2021;37(3):1025–41. 10.1007/s10899-021-10055-w 34255242

[10] Polo AJ, Makol BA, Castro AS, Colón-Quintana N, Wagstaff AE, Guo S. Diversity in randomized clinical trials of depression: a 36-year review. Clin Psychol Rev. 2019;1(67):22–35. 10.1016/j.cpr.2018.09.004 30292439

[11] Stoll CRT, Izadi S, Fowler S, Philpott-Streiff S, Green P, Suls J, et al. Multimorbidity in randomized controlled trials of behavioral interventions: a systematic review. Health Psychol. 2019;38(9):831–9. 31045382 10.1037/hea0000726 PMC6983953

[12] Van Spall HGC, Toren A, Kiss A, Fowler RA. Eligibility criteria of randomized controlled trials published in high-impact general medical journals: a systematic sampling review. JAMA. 2007;297(11):1233–40. 17374817 10.1001/jama.297.11.1233

[13] Jean-Louis G, Seixas AA. The value of decentralized clinical trials: Inclusion, accessibility, and innovation. Science. 2024;385(6711):eadq4994. 10.1126/science.adq4994 39172847

[14] Borschmann R, Patterson S, Poovendran D, Wilson D, Weaver T. Influences on recruitment to randomised controlled trials in mental health settings in England: a national cross-sectional survey of researchers working for the Mental Health Research Network. BMC Med Res Methodol. 2014;14(1):23. 24533721 10.1186/1471-2288-14-23 PMC3928923

[15] Mason V, Shaw A, Wiles N, Mulligan J, Peters T, Sharp D, et al. GPs’ experiences of primary care mental health research: a qualitative study of the barriers to recruitment. Fam Pract. 2007;24(5):518–25. 17698979 10.1093/fampra/cmm047

[16] Bierer BE, Meloney LG, Ahmed HR, White SA. Advancing the inclusion of underrepresented women in clinical research. Cell Rep Med. 2022;3(4):100553. 35492242 10.1016/j.xcrm.2022.100553 PMC9043984

[17] Hughson J anne, Woodward-Kron R, Parker A, Hajek J, Bresin A, Knoch U, et al. A review of approaches to improve participation of culturally and linguistically diverse populations in clinical trials. Trials. 2016;17(1):263. 10.1186/s13063-016-1384-3 PMC4880985 27229153

[18] Evaluating inclusion and exclusion criteria in clinical trials. U.S. Food and Drug Adminstration. 2018:1–10. Available from: https://www.fda.gov/media/134754.

[19] Hanvey GA, Johnson H, Cartagena G, Dede DE, Krieger JL, Ross KM, et al. The role of social, economic, and medical marginalization in cancer clinical trial participation inequities: a systematic review. J Clin Transl Sci. 2025;9(1):e25. 40052046 10.1017/cts.2024.677 PMC11883616

[20] Rodríguez-Torres E, González-Pérez MM, Díaz-Pérez C. Barriers and facilitators to the participation of subjects in clinical trials: an overview of reviews. Contemp Clin Trials Commun. 2021;23:100829. 10.1016/j.conctc.2021.100829 PMC8358641 34401599

[21] Webb Hooper M, Asfar T, Unrod M, Dorsey A, Correa JB, Brandon KO, et al. Reasons for exclusion from a smoking cessation trial: an analysis by race/ethnicity. Ethn Dis. 2019;29(1):23–30. 30713413 10.18865/ed.29.1.23 PMC6343546

[22] Humphreys K, Weisner C. Use of exclusion criteria in selecting research subjects and its effect on the generalizability of alcohol treatment outcome studies. AJP. 2000;157(4):588–94. 10.1176/appi.ajp.157.4.588 10739418

[23] Adams-Campbell LL, Ahaghotu C, Gaskins M, Dawkins FW, Smoot D, Polk OD, et al. Enrollment of African Americans onto clinical treatment trials: study design barriers. JCO. 2004;22(4):730–4. 10.1200/JCO.2004.03.160 14966098

[24] Bastien CH, Vallières A, Morin CM. Validation of the Insomnia Severity Index as an outcome measure for insomnia research. Sleep Med. 2001;2(4):297–307. 11438246 10.1016/s1389-9457(00)00065-4

[25] VanderWeele TJ, Robinson WR. On causal interpretation of race in regressions adjusting for confounding and mediating variables. Epidemiology. 2014;25(4):473–84. 24887159 10.1097/EDE.0000000000000105 PMC4125322

[26] Cohen AK, Ryan S, Smith LH, Ream RK, Glymour MM, Lopez A, et al. Educational attainment past the traditional age of completion for two cohorts of US adults: inequalities by gender and race/ethnicity. Race Soc Probl. 2022;14(3):208–22.

[27] Sparkman R, Tillman KH. Household income by nativity status and race/ethnicity across metropolitan and regional contexts. Popul Res Policy Rev. 2024;43(1):6.

[28] Murthy VH, Krumholz HM, Gross CP. Participation in cancer clinical trialsrace-, sex-, and age-based disparities. JAMA. 2004;291(22):2720–6. 15187053 10.1001/jama.291.22.2720

[29] Strait A, Castillo F, Choden S, Li J, Whitaker E, Falasinnu T, et al. Demographic characteristics of participants in rheumatoid arthritis randomized clinical trials: a systematic review. JAMA Netw Open. 2019;2(11):e1914745. 31722023 10.1001/jamanetworkopen.2019.14745 PMC6902779

[30] Filbey L, Zhu JW, D’Angelo F, Thabane L, Khan MS, Lewis E, et al. Improving representativeness in trials: a call to action from the Global Cardiovascular Clinical Trialists Forum. Eur Heart J. 2023;44(11):921–30. 36702610 10.1093/eurheartj/ehac810 PMC10226751

[31] Schwartz RC. Racial disparities in psychotic disorder diagnosis: a review of empirical literature. World J Psychiatr. 2014;4(4):133. 25540728 10.5498/wjp.v4.i4.133 PMC4274585

[32] Williams DR, Mohammed SA. Discrimination and racial disparities in health: evidence and needed research. J Behav Med. 2009;32(1):20–47. 19030981 10.1007/s10865-008-9185-0 PMC2821669

[33] Britton LE, Berry DC, Hussey JM. Comorbid hypertension and diabetes among U.S. women of reproductive age: prevalence and disparities. J Diabetes Complications. 2018;32(12):1148–52. 30291018 10.1016/j.jdiacomp.2018.09.014 PMC6289742

[34] Parra A, Morales V, Lebron CN, Santos HP Jr. Health implications of Black identity among Latinos: a call for Afro-Latina representation in maternal child health research. Nurs Res. 2024;73(6):417–9. 39466780 10.1097/NNR.0000000000000769 PMC12206385

[35] Lebron CN, Mitsdarffer M, Parra A, Chavez JV, Behar-Zusman V. Latinas and maternal and child health: research, policy, and representation. Matern Child Health J. 2023. Available from: https://link.springer.com/10.1007/s10995-023-03662-z. Cited 2025 Feb 21. 10.1007/s10995-023-03662-z PMC10560314 37029892

[36] Cuevas AG, Dawson BA, Williams DR. Race and skin color in Latino health: an analytic review. Am J Public Health. 2016;106(12):2131–6. 27736206 10.2105/AJPH.2016.303452 PMC5104999

[37] Morgan LB, Rodriquez EJ, Juarez JJ, Pérez-Stable EJ. Black race matters in the Latino population. Am J Public Health. 2024;114(3):270–5. 38382028 10.2105/AJPH.2023.307452 PMC10882392

[38] Parchem JG, Gupta M, Chen HY, Wagner S, Mendez-Figueroa H, Chauhan SP. Adverse infant and maternal outcomes among low-risk term pregnancies stratified by race and ethnicity. Obstet Gynecol. 2020;135(4):925. 32168221 10.1097/AOG.0000000000003730

[39] Valerio VC, Downey J, Sgaier SK, Callaghan WM, Hammer B, Smittenaar P. Black-White disparities in maternal vulnerability and adverse pregnancy outcomes: an ecological population study in the United States, 2014–2018. Lancet Reg Health – Am. 2023;20. Available from: https://www.thelancet.com/journals/lanam/article/PIIS2667-193X%2823%2900030-3/fulltext?stream=top. Cited 2025 Mar 4. 10.1016/j.lana.2023.100456 PMC10122115 37095772

[40] Jahan N, Went TR, Sultan W, Sapkota A, Khurshid H, Qureshi IA, et al. Untreated depression during pregnancy and its effect on pregnancy outcomes: a systematic review. Cureus. 2021;13(8):e17251. 34540477 10.7759/cureus.17251 PMC8448270

[41] Ferguson JM, Bradshaw PT, Eisen EA, Rehkopf D, Cullen MR, Costello S. Distribution of working hour characteristics by race, age, gender, and shift schedule among U.S. manufacturing workers. Chronobiol Int. 2023;40(3):310–23. 10.1080/07420528.2023.2168200 PMC10192004 36691907

[42] Olfson M, Marcus SC. National patterns in antidepressant medication treatment. Arch Gen Psychiatry. 2009;66(8):848–56. 19652124 10.1001/archgenpsychiatry.2009.81

[43] Yamamoto A, McCormick MC, Burris HH. Disparities in antidepressant use in pregnancy. J Perinatol. 2015;35(4):246–51. 25411773 10.1038/jp.2014.197 PMC4380708

[44] Givens JL, Houston TK, Van Voorhees BW, Ford DE, Cooper LA. Ethnicity and preferences for depression treatment. Gen Hosp Psychiatry. 2007;29(3):182–91. 17484934 10.1016/j.genhosppsych.2006.11.002

[45] Lara MaA, Navarrete L, Nieto L, Berenzon S. Acceptability and barriers to treatment for perinatal depression. An exploratory study in Mexican women. Salud Ment. 2014;37(4):293–301.

[46] Turner EA, Cheng HL, Llamas JD, Tran AGTT, Hill KGTT, Fretts J, et al. Factors impacting the current trends in the use of outpatient psychiatric treatment among diverse ethnic groups. CPSR. 2016;12(2):199–220.

[47] Vargas SM, Cabassa LJ, Nicasio A, De AA, Cruz L, Jackson E, et al. Toward a cultural adaptation of pharmacotherapy: Latino views of depression and antidepressant therapy. Article Transcultural Psychiatry. 2015;52(2):244. 25736422 10.1177/1363461515574159

[48] Louie CSY, Liu WM. Review of handbook of adult psychopathology in Asians. Cult Divers Ethn Minor Psychol. 2014;20(1):139–40.

[49] Nandagiri V, Vannemreddy S, Spector A. Sleep disparities in Asian Americans: a comprehensive review. J Clin Sleep Med. 2023;19(2):393–402. 10.5664/jcsm.10330 PMC9892749 36239044

[50] Sue S, Yan Cheng JK, Saad CS, Chu JP. Asian American mental health: a call to action. Am Psychol. 2012;67(7):532–44. 23046304 10.1037/a0028900

[51] Cheng AW, Chang J, O’Brien J, Budgazad MS, Tsai J. Model minority stereotype: influence on perceived mental health needs of Asian Americans. J Immigr Minor Health. 2017;19(3):572–81. 27246287 10.1007/s10903-016-0440-0

[52] Otte JL, Bakoyannis G, Rand KL, Ensrud KE, Guthrie KA, Joffe H, et al. Confirmatory factor analysis of the Insomnia Severity Index (ISI) and invariance across race: a pooled analysis of MsFLASH data. Menopause. 2019;28(8):850–5. 10.1097/GME.0000000000001343 PMC6663566 30994570

[53] Felder JN, Epel ES, Neuhaus J, Krystal AD, Prather AA. Efficacy of digital cognitive behavioral therapy for the treatment of insomnia symptoms among pregnant women: a randomized clinical trial. JAMA Psychiat. 2020;77(5):484–92. 10.1001/jamapsychiatry.2019.4491 PMC6990703 31968068

[54] Sordo Vieira L, Nguyen B, Nutley SK, Bertolace L, Ordway A, Simpson H, et al. Self-reporting of psychiatric illness in an online patient registry is a good indicator of the existence of psychiatric illness. J Psychiatr Res. 2022;1(151):34–41. 10.1016/j.jpsychires.2022.03.022 PMC10024540 35436704

[55] Ponting C, McClelland B, Mah R, Neuhaus J, Manber R, Krystal AD, et al. Effects of recruitment messaging on ethnic/racial minority screening in a RCT for Prenatal insomnia: an experimental approach. Behav Sleep Med. 2025;5:1–10. 10.1080/15402002.2025.2473346 PMC12045717 40040476

